# Perils, pitfalls and potential for the use of reporting guidelines in experimental research in medical education

**DOI:** 10.1007/s40037-019-00526-7

**Published:** 2019-07-19

**Authors:** Alice Moult, Peter Yeates

**Affiliations:** Medical School Education Research Group (MERG), Keele University School of Medicine, David Weatherall Building, Keele University, Keele, Staffordshire UK

In this issue, Kremer et al. [1] present a thoughtful study which experimentally examines the effects of negative emotions on medical residents’ learning. Within the paper, the authors provide a detailed and thorough description of their methods, yet chose not to follow a reporting guideline when writing-up their study. Whilst there are some examples of the use of reporting guidelines in medical education research [2], it is not common practice. In this commentary we aim to discuss some of the potential benefits and pitfalls for our field of more widespread adoption of reporting guidelines for experimental research.

The most established reporting guideline for trials is the Consolidated Standards of Reporting Trials (CONSORT) 2010 statement [3]. This is a 25 item checklist accompanied by a flow diagram. It provides guidance for reporting all randomized controlled trials, but focuses on the most common design: individually randomized, two group, parallel trials. CONSORT is endorsed by prominent general medical journals, specialty journals and leading editorial organizations [4]. Other reporting guidelines also exist (for example the American Psychological Association Journal Article Reporting Standards [5], and the American Educational Research Association Standards for Reporting Empirical Social Science Research [6]), but are less explicit in their recommendations. Kremer et al. described their study as a two group experiment, but the research shares many similarities with a randomized controlled trial, namely the randomization of participants to an experimental or control group, the measurement of outcomes for both groups, and the specific comparison of each group through statistical analyses. If Kremer et al. had followed the CONSORT statement, this might have provided further clarification to their description of the design, and any changes made to the design once the study had commenced.

CONSORT focuses on six key areas: the title and abstract, introduction, methods, results, discussion and other information. A number of items within these topics were addressed by Kremer et al., for example: the study is grounded in a clear theoretical background; explains the methods (including the experimental manipulation) thoroughly; and provides details about the statistical methods used to compare the groups. Other items, such as eligibility criteria for participants, how the sample size was determined and the type of randomization (e.g. blocking or block size) were less clearly described. Perhaps most valuably, the use of CONSORT would have encouraged an unequivocal description of the journey of different participants: how many were screened, how many randomized, how many were allocated to each, whether they all received their intervention as intended, and details of any who dropped out or whose data were not included in analyses. Use of the CONSORT statement might have further enhanced the clarity, transparency and completeness of reporting, and in turn helped to ensure replicability.

We believe that reporting guidelines are beneficial and their use should be encouraged as they permit editors, peer reviewers and readers to better understand studies. However, whilst there may be benefits for the field of medical education in adopting reporting guidelines, they are not without their critics. Authors may feel like they have to shoehorn their studies into a mould which may not work for the context or research questions. This may be particularly problematic for qualitative research which is divergent in theoretical and methodological assumptions about the nature of the phenomena being explored and how it can, and should be, researched [7, 8]. Experimental studies derive from a more singular conception of validity, and so the benefits of reporting guidelines for consistency and clarity are easier to appreciate. Nonetheless, some queries may be raised about the applicability of the CONSORT guidelines for experiments in medical education which are, after all, not clinical trials. Typically, educational experiments or trials are conducted over briefer periods of time than clinical trials, and so some of the features of CONSORT (for example interim analyses and stopping rules) are unlikely to apply. Furthermore, experiments may contain features which CONSORT does not describe. For example, Kremer et al. used a deceptive premise and subsequent debrief which they conducted and described well [1]. CONSORT would not have guided them in this procedure. Reporting guidelines encourage authors to report how they calculated their sample size. The concept of power is challenging in the field of medical education as there are often no prior studies to prospectively power studies from. Nonetheless, a comparatively uniform approach to reporting statistical power could still be advocated; perhaps one which requires researchers to state their minimally important difference and a retrospective analysis of the power they had to detect that difference. Consequently, adaptation of this guidance may be required for it to align with the type of experiments which are typical in medical education.

In conclusion, to both support methodological rigour and clarify reporting, we advocate for the use of reporting guidelines for experimental studies in medical education. Before this can occur, researchers in the field of medical education may need to have a wider discussion regarding which guideline is most appropriate, or if we should consider developing our own.
